# Transforming Growth Factor-β Signaling in Alcohol-Associated Liver Disease: A Multicellular Perspective

**DOI:** 10.1016/j.ajpath.2025.09.017

**Published:** 2025-10-16

**Authors:** Huihui Zou, Sai Wang, Chenjun Huang, Steven Dooley, Nadja M. Meindl-Beinker

**Affiliations:** ∗ Section Molecular Hepatology, Department of Medicine II, Medical Faculty Mannheim, Heidelberg University, Mannheim, Germany; † Department of Medicine II, Medical Faculty Mannheim, Heidelberg University, Mannheim, Germany

## Abstract

Transforming growth factor-β (TGF-β) signaling exerts broad regulatory effects on alcohol-associated liver disease (ALD) progression, influencing processes such as hepatocellular injury, regeneration, inflammation, fibrogenesis, cirrhosis, carcinogenesis, and hepatic failure. TGF-β modifies alcohol-induced signals in multiple liver-resident cell types, including hepatocytes, hepatic stellate cells, liver sinusoidal endothelial cells, and immune populations, particularly macrophages. To delineate its context-specific roles in ALD, 154 of 421 PubMed-listed publications (2000 to 2025; search terms TGF-β and alcohol and liver disease) were reviewed, supplemented by 19 foundational studies published earlier. In hepatocytes, TGF-β promotes oxidative stress, apoptosis, metabolic reprogramming, and epithelial-to-mesenchymal transition. In hepatic stellate cells and Kupffer cells, gut-derived endotoxins, ethanol, and unsaturated fatty acids induce TGF-β alongside proinflammatory cytokines. Ethanol metabolism generates acetaldehyde, which drives TGF-β and receptor expression, enhances canonical and noncanonical signaling, and engages epigenetic regulators to promote extracellular matrix deposition. In liver sinusoidal endothelial cells, alcohol-induced TGF-β suppresses proliferation, contributing to sinusoidal capillarization, impaired endothelial regeneration, and fibrogenesis. TGF-β dampens clearance of damaged hepatocytes and perpetuating chronic injury by suppressing natural killer cell cytotoxicity and promoting regulatory T-cell differentiation. At end-stage disease, TGF-β promotes expansion and fate switching of cholangiocyte-derived liver progenitor cells to replenish lost hepatocytes. Despite its central role in ALD, therapeutic exploitation of TGF-β signaling remains underexplored. Future studies should define cell type–specific signaling nodes to enable precision therapies.

According to the Global Burden 2023 Update, liver disease accounts for approximately 2 million deaths annually, representing 4% of global mortality. ^1^ Alcohol consumption is a major risk factor for various liver diseases and remains widespread globally.^2^ Importantly, not alcohol alone, but rather its combination with a range of additional factors, including age, sex, and ethnicity, but also metabolic disorders influence the development and progression of alcohol-associated liver disease [also referred to as alcohol-related liver disease or alcoholic liver disease (ALD)].^2^ Thus, the spectrum of steatotic liver disease has been adjusted over recent years, attributing the term ALD to those patients only with a daily alcohol intake that exceeds 50 g for women and 60 g for men. Steatotic liver disease associated with less alcohol intake was renamed to metabolic dysfunction and alcohol-related liver disease (MetALD; 20 to 50 or 30 to 60 g of alcohol per day for women and men, respectively) or metabolic dysfunction–associated steatotic liver disease (<20 or 30 g of alcohol per day). As obesity and metabolic syndrome–associated complications are on the rise worldwide, with a prevalence of nonalcoholic fatty liver disease up to 32.4%,^1^ thus affecting an extraordinary vast number of patients, the need for respective medications seems obvious. Newly developed and costly drugs, such as glucagon-like peptide-1 receptor agonists, but also other alternatives, meet this high demand and may help a vast number of patients. However, it should be kept in mind that alcohol is the far more dangerous liver disease driver per se.

ALD usually develops from simple steatosis (alcohol-related fatty liver disease) to more severe forms, such as steatohepatitis, fibrosis, cirrhosis, end-stage liver disease [in the case of alcohol, it is termed alcohol-related hepatitis (AH)], and hepatocellular carcinoma (HCC).^3^ Alcohol-related fatty liver disease is characterized by the accumulation of lipid droplets within hepatocytes. This early stage is generally reversible with alcohol cessation, although a small proportion of cases may still progress to fibrosis and beyond.^4^

If alcohol consumption persists, ALD may progress to fibrosis, including connective tissue deposition and cirrhosis.^4^^,^^5^ Cirrhosis is characterized by extensive liver tissue scarring and formation of regenerative nodules.^6^ Severe AH develops after years of heavy drinking, presenting with a sudden onset of jaundice and rapid liver decompensation,^7^^,^^8^ histologically characterized by the presence of macrovesicular steatosis, infiltration of leukocytes, swelling of hepatocytes (ballooning degeneration), and the formation of Mallory-Denk bodies.^9^ Severe AH is a critical complication of ALD characterized by profound hepatocellular dysfunction, and as many as 40% of affected patients die within 6 months of the onset of symptoms.^9^ New targeted treatments of this life-threatening condition are urgently needed.^10^ The disease progression rate varies individually, influenced by the complex interplay of genetic, environmental, and other factors, numbering in 50% of heavy drinkers advancing to cirrhosis.^2^^,^^11^^,^^12^

Many studies have also delineated the indirect liver inflammatory signals of alcohol effects on the gut microbiota, altering its composition, gut leakiness, and increased levels of endotoxins, such as lipopolysaccharide (LPS), that directly affect the liver cells.^13^

Transforming growth factor-β (TGF-β) is a key cytokine involved in multiple cellular processes and cell fate decisions, including cell growth control, differentiation, immune regulation, fibroblast activation, angiogenesis, and epithelial-to-mesenchymal transition (EMT) in cancer progression.^14^^,^^15^ In the liver, TGF-β is especially known as one of the major drivers of liver fibrosis, a key process in almost all chronic liver diseases (CLDs), including ALD.^16^^,^^17^ In this review article, we will elaborate on TGF-β signaling pathways in the liver and more specifically what is known in ALD with a special emphasis on cell type–specific TGF-β effects. This review exclusively comprises knowledge on TGF-β, knowing that the TGF-β ligand family consists of 33 members, including also activins, bone morphogenic proteins (BMPs), and growth and differentiation factors, which as well have been described as relevant in liver (patho-)physiology.^18^

## Regulating Fibrosis and Beyond: TGF-β Signaling in the Liver

In ALD, TGF-β signaling emerges as a central orchestrator of fibrogenic progression, triggered by both ethanol itself and hepatocyte-derived signals. Alcohol metabolism in hepatocytes produces acetaldehyde, reactive oxygen species (ROS), and damage-associated molecular patterns that sensitize neighboring hepatocytes, nonparenchymal cells, and the extracellular matrix (ECM) to TGF-β activation and signaling, and promote its synthesis in Kupffer cells (KCs), hepatic stellate cells (HSCs), and liver sinusoidal endothelial cells (LSECs).^7^^,^^19^, ^20^, ^21^ Following its transcription and translation, TGF-β in every cell type undergoes complicated but essential post-translational modifications that dictate its maturation and bioavailability. Briefly, the propeptide is cleaved by furin-like convertases in the Golgi to generate the latency-associated peptide and mature TGF-β dimer, stabilized by disulfide bonds and N-glycosylation.^22^^,^^23^ The large latent complex, consisting of latency-associated peptide–TGF-β bound to latent TGF-β–binding proteins (LTBPs), is secreted after further hydroxylation and glycosylation and tethered to the ECM, where it remains sequestered by extracellular matrix protein 1 (ECM1) in its latent form until activated by proteases, integrins, thrombospondins, or ROS-induced conformational changes.^24^, ^25^, ^26^ Once liberated, active TGF-β engages canonical Smad2/3 and noncanonical signaling cascades to drive HSC activation, LSEC capillarization, ECM deposition, hepatocyte apoptosis, and other mechanisms, which overall leads to liver disease progression, including ALD.^14^^,^^27^

Beyond biochemical maturation, the fibrogenic potency of TGF-β is amplified by intricate crosstalk among parenchymal and nonparenchymal cells. Briefly, injured hepatocytes release apoptotic bodies and damage-associated molecular patterns that activate KCs, which, in turn, secrete TGF-β, tumor necrosis factor (TNF)-α, and platelet-derived growth factor, mediating HSC activation, including proliferation, migration, and ECM production.^19^^,^^28^^,^^29^ HSCs are further sensitized to TGF-β through LPS–toll-like receptor 4 signaling, which down-regulates the inhibitory pseudoreceptor BMP and activin membrane-bound inhibitor (BAMBI).^30^ LSECs, normally maintaining HSC quiescence, lose their protective phenotype under ethanol exposure and secrete angiocrine factors that synergize with TGF-β in promoting HSC activation and fibrogenesis.^31^ Additionally, infiltrating immune cells, particularly T cells and monocyte-derived macrophages, produce cytokines, such as IL-17 and osteopontin, that reinforce TGF-β signaling in HSCs.^32^ These multidirectional communications form a feed-forward loop, where parenchymal injury fuels nonparenchymal activation, and the resulting profibrotic microenvironment further compromises hepatocyte viability (see also the compartment model; online Supplemental Figure S8 in the study by Link et al^25^).

**Figure 1:**
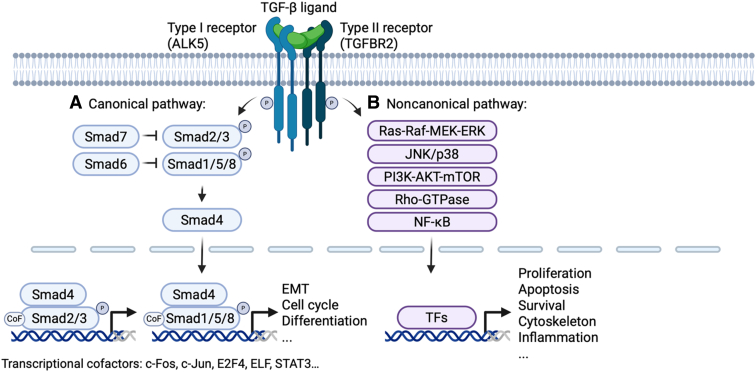
Schematic overview of transforming growth factor (TGF)-β signaling pathways. **A:** Canonical (Smad-dependent) pathway: TGF-β ligands are secreted in a latent, inactive form. On activation, they bind to the transmembrane type II receptor (TGFBR2), inducing its autophosphorylation and recruitment of the type I receptor kinase (ALK5). The resulting ligand-receptor complex phosphorylates receptor-regulated Smads (Smad2/3 or Smad1/5/8), which form hetero-oligomers with the common mediator Smad4. These complexes translocate to the nucleus and, with cofactors (eg, c-Fos, c-Jun, E2F4, and STAT3), regulate target gene transcription. Inhibitory Smads (Smad6/7), themselves transcriptional targets of Smad signaling, provide negative feedback. **B:** Noncanonical (Smad-independent) pathways: TGF-β receptors also activate alternative signaling cascades, including RAS–Raf-1 proto-oncogene, serine/threonine kinase (RAF)–mitogen-activated protein kinase kinase (MEK)–extracellular signal-regulated kinase (ERK), c-Jun N-terminal protein kinase (JNK)/p38 mitogen-activated protein kinase, phosphatidylinositol 3-kinase (PI3K)–AKT–mammalian target of rapamycin (mTOR), Rho GTPases, and NF-κB, modulating TGF-β signaling and diverse cellular responses. The figure was generated in BioRender.com (Toronto, ON, Canada). EMT, epithelial-to-mesenchymal transition; TF, transcription factor.

Following TGF-β synthesis, maturation, and activation, canonical signaling of the TGF-β ligand is well studied and comprises five consequential steps (Figure 1A).^33^^,^^34^ These are receptor binding by TGF-β ligands, receptor homodimerization and heterodimerization, phosphorylation and activation of receptor- and co-Smads, and Smad complex translocation to the nucleus where tissue-, cell type–, and context-dependent transcription regulation is orchestrated by the assembly of a respective and variant transcriptional complex.

Signaling is induced by binding of the ligands to type II TGF-β receptor (TβRII or TGFBR2) molecules and followed by recruitment of type I TGF-β receptors (TβRIs or TGFBR1s), leading to the formation of heterotetrameric complexes.^35^ The TGF-β receptors, a family of seven type I and five type II receptors, are dual specificity receptors comprising serine, threonine, and tyrosine kinase activities.^36^^,^^37^ On complex formation, TGFBR2 induces TβRI phosphorylation at different sites, activating further signal transduction. The ligand-activated receptor complex then leads to subsequent phosphorylation of the intracellular mediators of TGF-β signaling, the receptor (R)-Smad proteins. Generally, there are two R-Smad signaling branches (ie, the Smad2/3 and the Smad1/5/8 pathway).^38^^,^^39^ Activated R-Smad proteins of either branch dissociate from the receptor complex and interact with common-Smad4, which then translocates into the nucleus with prolonged retention time in that compartment.^40^^,^^41^ In the nucleus, transcription of several hundred target genes is regulated by the Smad complexes in cooperation with different transcription factors, co-activators, and corepressors.^42^, ^43^, ^44^, ^45^ Interestingly, dependent on the liver physiological or pathophysiological stage, TGF-β signaling can exhibit variant functions in the same cells, including tumor-suppressor or tumor-promoter effects in epithelial cells.^46^

TGF-β signaling is tightly controlled on multiple levels, from intracellular synthesis and processing, secretion and ECM deposition, activation of the latent form, receptor complex formation, intracellular propagation of the signaling and hierarchical interaction with activating and inhibitory cofactors that lead to signaling component degradation, or selection of target gene DNA binding. Zooming further on signaling regulation would exceed the scope of this review article, and can instead be read in other review articles specifically focusing on TGF-β signaling regulation, among others, Taylor and Wrana,^47^ or more recent ones by Heldin and Moustakas^35^ or Derynck and Budi.^34^

Smad6 and Smad7 are well-characterized inhibitory Smads, with Smad6 being specific to Smad1/5/8 signaling and Smad7 being able to inhibit both canonical branches at a different level of the signaling process by binding to the respective TβRI molecules.^39^^,^^48^ As *Smad7* is a direct TGF-β target gene, it provides an important and efficient regulatory negative feedback loop.^49^ Furthermore, Smad7 is characterized as a molecular switch contributing to the determination of whether TGF-β signaling has protumorigenic or antitumorigenic effects.^50^^,^^51^

Other inhibitory mechanisms have been identified, acting to terminate TGF-β signaling. Besides receptor dephosphorylation, receptor and Smad protein ubiquitinylation and subsequent proteasomal degradation are described.^52^

Noncanonical TGF-β signaling involves TGF-β receptor complex-mediated mitogen-activated protein kinase (MAPK), extracellular signal-regulated kinase (ERK), c-Jun N-terminal protein kinase (JNK), p38, and phosphatidylinositol 3-kinase–AKT pathway activation, which are frequently used for fine-tuning of TGF-β effects (Figure 1B) and which can be studied in detail in the review articles of Deng et al^53^ or Zhang.^54^ Briefly, ERK MAPK signaling activation usually occurs quickly on ligand binding to TGF-β receptors and phosphorylation of TβRI at its tyrosine residues.^55^ It is dependent on Src homology and collagen A adaptor protein (ShcA) phosphorylation, leading to complexation with growth factor receptor-bound protein 2 (Grb2) and son of sevenless (SOS). This, in turn, activates Ras and the downstream Raf/mitogen-activated protein kinase kinase (MEK) cascade. TGF-β–induced ERK signaling may also regulate a variety of cellular responses, including TGF-β–dependent EMT. Interestingly, TGF-β–mediated ERK/MAPK activation leads to different responses compared with receptor–tyrosine kinase–mediated ERK/MAPK signaling.

Next, TGF-β may regulate JNK and p38 signaling via recruiting tumor necrosis factor receptor associated factor (TRAF) 4 and TRAF6, leading to activation of TGF-β–activated kinase (TAK) 1. This leads to activation of Rho GTPases, such as ras homolog family member A (RHOA), ras-related C3 botulinum toxin substrate 1 (RAC1), and cell division control protein 42 homolog (CDC42), which further facilitate p38 and JNK activation.^53^ TGF-β/TRAF6 activation can also result in phosphatidylinositol 3-kinase–mediated signal transduction with phosphorylation of phosphatidylinositol 4,5-bisphosphate (PIP2) and AKT.

Importantly, TRAF/TAK1, RHO/Rho-associated protein kinase (ROCK), and phosphatidylinositol 3-kinase/AKT signaling pathways can converge in activation of nuclear factor kappa-light-chain-enhancer of activated B cells (NFxB), which is supporting cell survival, proliferation, an inflammatory phenotype and metabolic activity. In HSCs, TGF-β has additionally been reported to be able to activate JAK/STAT signaling (Figure 1).^56^^,^^57^

Overall, noncanonical TGF-β signaling adds substantial complexity to liver physiology and pathology by integrating canonical and other signaling pathways to fine-tune cellular responses, thereby orchestrating the liver's responses to the diverse challenges associated with the onset and progression of CLD, including ALD.

## TGF-β Expression in ALD

The presence of alcohol changes in liver gene expression signatures, including proinflammatory and profibrogenic cytokines, such as TGF-β.^58^^,^^59^ TGF-β expression up-regulation and increased signaling activation were described in the context of elevation of gut-derived endotoxins, increases in oxidative stress, and accumulation of ethanol metabolites, such as acetaldehyde and lipid oxidation products, all as prerequisites of hepatocellular damage, as well as HSC and KC activation.^60^, ^61^, ^62^ Its up-regulation was also consistently found in advanced fibrosis and cirrhosis of patients with ALD.^2^^,^^17^^,^^60^, ^61^, ^62^, ^63^, ^64^, ^65^, ^66^, ^67^

In a clinical study including of 30 patients with ALD, divided into four groups along clinical findings and liver histology, that are i), steatosis; ii), fibrosis; iii), hepatitis; and iv), cirrhosis, liver biopsies were tested for *TGFB1, TGFB2*, and *TGFB3* gene expression by RT-PCR. Increasing expression of all isoforms was found with severity of the ALD, whereby differences exist among the isoforms.^63^

In another cohort of 107 male patients with different stages of ALD, peripheral blood mononuclear cells were analyzed for TGF-β mRNA expression. Patients were grouped into alcohol abusers without liver impairment (*n* = 22), alcohol-related steatosis (*n* = 30), alcohol-related hepatitis (*n* = 31), and alcohol-related cirrhosis (*n* = 24). A total of 34 healthy subjects served as control. Expression of TGF-β from all patients with ALD with a liver pathology was higher than in controls. Differences of expression levels were significant between the patients from each group, whereby alcohol abusers without liver impairment did not differ from controls. The study is providing some evidence that TGF-β expression levels in peripheral blood mononuclear cells are indicative for alcohol-associated liver disease and may refer to inflammatory activity and fibrosis in the liver.^2^ Interestingly, it is also reported that in patients with compensated and decompensated alcohol-related cirrhosis, TGF-β levels in the serum were comparable to controls.^16^

Functional evidence for TGF-β as driver in ALD was provided from data with hepatocyte-specific deletion of Smad7 in mice, which results in aggravated alcohol-induced liver injury and fibrosis.^6^^,^^16^^,^^68^ Chen and coworkers^68^ fed mice with a liquid diet containing 5% ethanol for 6 weeks, followed by a single dose of ethanol gavage, to mice depleted for Smad7 in hepatocytes (Smad7ΔHep). As a consequence, Smad2/3 phosphorylation was spontaneously increased in isolated hepatocytes, which was further enhanced on TGF-β treatment. Alcohol-induced liver injury and steatosis were profoundly aggravated in Smad7ΔHep mice and associated with up-regulation of genes involved in lipogenesis and inflammation, whereas alcohol dehydrogenase 1 (ADH1) expression was abrogated (more detail, see below). Approximately 30% of these Smad7ΔHep mice presented with spontaneous liver dysfunction, including low body weight, overall deterioration, and elevated hepatocyte apoptosis, with increased serum aspartate aminotransferase and alanine aminotransferase levels. Furthermore, EMT of cultured hepatocytes from these mice was accelerated, compared with controls, when treated with TGF-β.

In mice fed with ethanol-containing L. De Carli diets, TGF-β and BMP2 expression are up-regulated; this resulted only in increased Smad2 phosphorylation, whereas no change to controls can be found for BMP receptor activation or Smad1 and Smad5 phosphorylation.^61^ In the same setting, Smad4 DNA-binding activity to the hepcidin promoter is attenuated, leading to the conclusion that alcohol integrates on TGF-β/BMP/Smad signaling in a way that compromises Smad complex-mediated hepcidin transcription and iron metabolism with impact on liver injury, probably by redirecting the canonical pathway toward other target genes.

In mice, it was shown that chronic ethanol feeding influenced eight canonical pathways in the liver, including tight junction signaling, pulmonary fibrosis idiopathic signaling pathway, integrin-linked kinase signaling, hepatic fibrosis signaling, Wnt/β-catenin signaling, clear signaling, TGF-β1 signaling, production of nitric oxide and ROS in macrophages, phosphatase and tensin homolog (PTEN) signaling, and inhibitor of differentiation.^69^ The TGF-β–mediated anti-proliferative and pro-apoptotic change in hepatocyte characteristics under certain conditions and the HSC activating capacity of this cytokine are discussed in more detail below.

Regarding susceptibility to ALD, several polymorphisms were identified as risk factors. In that line, polymorphisms in the *TGFB1* gene were analyzed in 165 patients with advanced ALD versus 185 healthy controls, where no correlation to the risk for advanced ALD was found.^70^ To draw final conclusions in that matter, however, a much larger group^71^ of patients and controls needs to be investigated.

**Figure 2:**
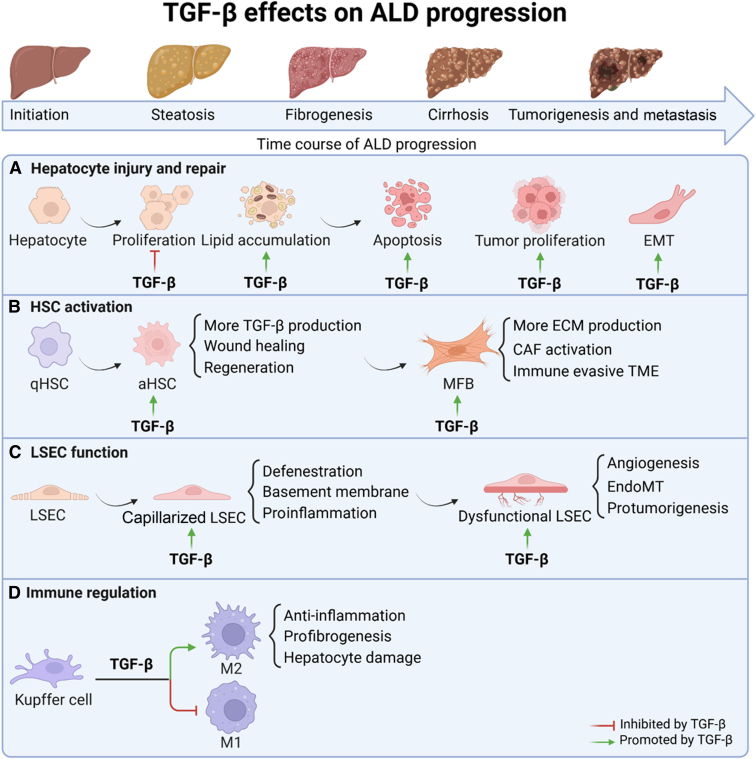
Cell type–specific roles of transforming growth factor (TGF)-β in alcohol-associated liver disease (ALD) pathogenesis. Schematic overview of TGF-β’s pleiotropic effects across hepatic cell populations. **A:** Hepatocytes: TGF-β drives apoptosis, epithelial-to-mesenchymal transition (EMT), and metabolic dysfunction. **B:** Hepatic stellate cells (HSCs): TGF-β directs activation, fibrogenesis (∗COL1A1/3∗, *ACTA2*), and autocrine TGF-β amplification. **C:** Liver sinusoidal endothelial cells (LSECs): TGF-β induces capillarization via cell cycle arrest and phenotypic reprogramming. **D:** Immune regulation: TGF-β modulates Kupffer cell polarization, suppresses natural killer cell surveillance, and promotes type 17 helper T-cell/regulatory T-cell imbalance. The figure was generated with BioRender.com (Toronto, ON, Canada). aHSC, activated hepatic stellate cell; CAF, cancer associated fibroblast; ECM, extracellular matrix; endoMT, endothelial-to-mesenchymal transition; MFB, myofibroblast; qHSC, quiescent hepatic stellate cell; TME, tumor microenvironment.

In the following paragraphs, we will discuss the specific influence of TGF-β on the different liver cell types (ie, hepatocytes, HSCs, cholangiocytes, and immune cells, including KCs and LSECs) and what has been found in the context of ALD. A schematic overview of the following is depicted in Figure 2.

## Dual Insult: TGF-β and Ethanol Cooperate to Drive Hepatocellular Injury in ALD

TGF-β is well known to induce growth arrest on proliferating hepatocytes in the context of liver regeneration after injury.^16^^,^^60^^,^^72^^,^^73^

Cultured mouse hepatocytes spontaneously transdifferentiate, lose hepatocyte features, undergo EMT, and up-regulate survival signaling pathways, all of which is enhanced by TGF-β treatment. In parallel, TGF-β down-regulates multiple metabolic functions of hepatocytes. TGF-β treatment of cultured mouse hepatocytes enhances ethanol-induced oxidative stress, toxicity, and inflammatory response to endotoxins and induces lipid-, oxidative stress–, metabolism-, and fibrogenesis-related gene expression signatures.^30^^,^^74^, ^75^, ^76^

Ethanol pretreatment massively increases the pro-apoptotic TGF-β effect in primary mouse hepatocytes, making hepatocytes more prone to injury even at relatively low alcohol concentrations,^60^ which is also reflected in boosted pro-apoptotic gene signatures, and makes hepatocytes sensitive to injury, even at relatively low alcohol concentrations.^60^ When exposed to TGF-β in the presence of ethanol, hepatocytes even increase transcription of genes involved in lipid metabolism, oxidative stress, and fibrogenesis, indicating a shift in cellular fate toward dysfunction and damage.^74^ In both, chronic ethanol-fed mice and liver samples from patients with ALD, TGF-β signaling is also markedly activated.^74^

TGF-β signaling-mediated apoptosis is transmitted via canonical SMAD2/3 signaling, which induces downstream mediators, such as NADPH oxidase 4 and pro-apoptotic Bcl-2 family members Bcl-2 interacting mediator of cell death (BIM) and Bcl-2-modifying factor (BMF).^77^, ^78^, ^79^ Furthermore, ethanol sensitizes hepatocytes for TGF-β signaling by up-regulating TGFBR2 expression, which enhances receptor-mediated pro-apoptotic signaling of TGF-β.^60^ However, recent findings suggest that ethanol-dependent enhancement of the pro-apoptotic effects of TGF-β in hepatocytes even goes beyond what can be explained by canonical SMAD signaling alone.^60^

A subsequent in-depth investigation delineated how crosstalk with survival signaling pathways in hepatocytes is critical for the outcome of the TGF-β pathway, therewith also providing a first explanation on how rebranching of the pathway from cytostatic and tumor suppressive to tumor promoting may occur. Our own data show that activation of the AKT pathway and its downstream effector glycogen synthase kinase-3β interfere with pro-apoptotic and proliferation inhibitory effects of TGF-β and rebranch the pathway to growth factor and growth factor receptor production and EMT in cultured mouse hepatocytes.^80^ Alcohol treatment (like an AKT inhibitor) inhibits AKT activation, therewith resensitizing hepatocytes to cytostasis, cell stress, and apoptotic cell death.^60^ Interestingly in this study, alcohol metabolism via cytochrome P450 2E1 (CYP2E1) and ADH activities as well as the aggressive metabolite acetaldehyde did not mimic the alcohol effect on TGF-β signaling, suggesting an independent function of ethanol directly integrating into signaling pathways. The findings in cell culture were confirmed in an ALD mouse model and in liver slice cultures from patients with HCC.^60^

The integration of TGF-β signaling on survival signaling was also impressively shown with a mouse model of depleted expression of the survival factor Tak1.^81^^,^^82^ Levels of TGF-β, TGFBR2, and phosphorylated Smad2/3 were increased in Tak1ΔHep mice. The mice spontaneously developed liver fibrosis and inflammation by 1 month and HCC by 9 months. Hepatocytes from Tak1ΔHep mice were deficient for TGF-β–mediated activation of p38, c-Jun N-terminal kinase, or nuclear factor-B, whereas TGF-β–mediated cell death and phosphorylation of Smad2/3 were increased, compared with control hepatocytes. Blocking the Smad pathway or disruption of Smad4 inhibited TGF-β–mediated death of Tak1-deficient hepatocytes, as well as spontaneous liver injury, inflammation, fibrosis, and HCC. The data suggest that massive cell death mediated by TGF-β signaling in hepatocytes with depleted survival signaling may lead to tumorigenesis via inflammation-driven compensatory proliferation induction.

Lopa Mishra and her team^83^, ^84^, ^85^, ^86^, ^87^, ^88^, ^89^, ^90^, ^91^, ^92^, ^93^, ^94^ have conducted extensive research on the switch in TGF-β signaling outcomes in hepatocytes. They showed that signaling can change from tumor suppressive to tumor promoting, and that the availability of Smad3-associated cofactors is critical to this transition. One such transcriptional cofactor, the adaptor protein embryonic liver fodrin (ELF) or β2-spectrin (β2SP), is prominently involved in tumor-suppressor functions and cell fate decisions downstream of TGF-β/Smad3 signaling. β2SP is frequently mutated or down-regulated in gastrointestinal tumors, including those of the liver. This results in the mislocalization of Smad3/Smad4 complexes and loss of E-cadherin accumulation at cell-cell junctions, thereby impairing E-cadherin–β-catenin–dependent epithelial cell-cell adhesion. Additionally, β2SP induces up-regulation of cyclin D1, cyclin-dependent kinase 4, c-Myc, and mouse double minute 2 homolog (MDM2). It also promotes phosphorylation of retinoblastoma gene product and increased cellular sensitivity to DNA cross-linking agents.^83^, ^84^, ^85^, ^86^, ^87^ Such transformed TGF-β signaling outcome integrates with inflammatory pathways, especially IL-6/Stat3. This leads to the activation of TAK1–NF-κB survival signaling and expression of EMT markers Twist and Slug, which, in this setting, triggers hepatocyte fate transformation toward CD133^+^ cancer stem cells.^88^^,^^89^ The group also demonstrated that a reduction of β2SP availability in liver regeneration processes leads to increased numbers of activated liver progenitor cells and DNA damage sensitive hepatocytes, both facilitating cancer cell formation.^86^^,^^90^ Because ethanol-derived acetaldehyde induces DNA damage, especially the latter mechanism is highly relevant for patients with ALD. In a model of alcohol-mediated DNA damage, β2SP is critical for maintenance of genomic stability by supporting DNA repair through β2SP-dependent activation of Fanconi anemia complementation group D2 (Fancd2), a core component of the Fanconi anemia complex. Loss of β2SP leads to decreased Fancd2 levels and sensitizes β2SP mutants to DNA damage by ethanol treatment. This leads to phenotypes that are similar to those observed in animals lacking both aldehyde dehydrogenase 2 (ALDH2) and Fancd2 and resemble human fetal alcohol syndrome. In line, *Sptbn1*-deficient cells are hypersensitive to DNA cross-linking agents and have defective DNA double-stranded break repair, which can be rescued by ectopic Fancd2 expression. Moreover, Fancd2 transcription in response to DNA damage/TGF-β stimulation is regulated by the β2SP/mothers against decapentaplegic homolog 3 complex.^91^ The group of Mishra^92^ developed an alcohol-associated liver disease model that more rapidly develops steatosis, inflammation, and fibrosis by treating the mice with a chronic low dose of LPS while feeding them a Lieber DeCarli diet. When applied to β2SP^+/–^ or Smad3^+/–^ mice, this approach surprisingly led to aggressive T-cell lymphomas, whereas their liver phenotype was mild.^92^ Finally, β2SP reduction indicates that TGF-β/Smad3 signaling may have adverse impact on progression of fatty liver disease. Although the results were generated in Western diet feeding models, similar outcomes can also be expected in ALD. β2SP drives lipogenesis, fibrosis, and liver cancer development in metabolic dysfunction–associated steatotic liver disease through interaction with, and caspase-3–mediated cleavage of, sterol regulatory element-binding protein 1 (SREBP1). In contrast, hepatocyte-specific loss or siRNA therapy against β2SP protects mice from obesity, liver damage, and nonalcoholic steatohepatitis progression. In line, human metabolic-associated steatohepatitis samples show elevated β2SP.^93^ Metabolic dysfunction–associated steatotic liver disease and metabolic-associated steatohepatitis are worsened in people with reduced ALDH2 function, leading to aldehyde accumulation and toxic 4-hydroxynonenal (4-HNE) buildup. *ALDH2*-deficient mice recapitulate features of metabolic syndrome and metabolic-associated steatohepatitis with profibrotic and pro-oncogenic signaling. Mechanistic studies reveal that 4-HNE modifies the adaptor protein β2SP, driving aberrant TGF-β/SMAD3 activation. Therapeutic inhibition of spectrin beta chain, brain 1 (SPTBN1) mRNA with siRNA restores normal signaling, blocks metabolic-associated steatohepatitis and fibrosis, and improves glucose metabolism.^94^

A spatial analysis of liver tissue indicates that ethanol treatment–induced expression of TGF-β1 and CYP2E1 occurs in the centrilobular area.^75^ Accordingly, ALD is usually initiated in the centrilobular region of the liver. HepG2 E47 cells expressing human CYP2E1 were shown to be more sensitive to growth inhibition and enhanced apoptosis from TGF-β1 treatment. This was accompanied by enhanced production of ROS and a decline in glutathione levels. The enhanced TGF-β effect could be abrogated with the CYP2E1 inhibitor diallyl sulfide, suggesting a direct link between TGF-β and CYP2E1. Moreover, TGF-β1 decreased the mitochondrial membrane potential before developing toxicity, and TGF-β1–induced cell death could be prevented by trifluoperazine, an inhibitor of the mitochondrial membrane permeability transition. In line with these findings, TGF-β1 compromised cell viability in hepatocytes from pyrazole-treated rats with elevated levels of CYP2E1 stronger than in control hepatocytes. In conclusion, potentiation of TGF-β effects on cell stress and death by CYP2E1 may contribute to mechanisms of ALD.

It was further demonstrated that human hepatocytes exposed to ethanol are more sensitive toward damage from TGF-β by enhancing oxidative stress via up-regulation of NADPH oxidases.^95^ *In vitro*, 100 mmol/L ethanol and/or 5 ng/mL recombinant human (rh)TGF-β1 induced cellular damage in human hepatocytes, with 50% to 55% of total lactate dehydrogenase released after 72 hours. Co-incubation with ethanol and TGF-β enhanced the damage by inducing ROS formation and reducing cellular glutathione levels. In patients with ALD, metabolism of consumed alcohol in the liver also rapidly increases ROS production.

This TGF-β–ethanol interplay reveals a critical mechanism driving hepatocyte dysfunction in ALD and highlights the importance of considering hepatocyte-specific responses when evaluating therapeutic strategies. It also underlines the need for interventions targeting noncanonical pathways, such as the AKT/glycogen synthase kinase-3β axis, rather than SMAD signaling alone. Clinically, these findings support the recommendation that patients with preexisting liver fibrosis or CLD should abstain from alcohol, as even moderate intake can synergistically enhance TGF-β–induced hepatocellular apoptosis and accelerate disease progression.^62^

The reader should also be aware that although TGF-β can inhibit liver tumor initiation and early development by suppressing cell growth, inducing apoptosis, and inhibiting cytokine and chemokine expression initially, in later stages of HCC development, TGF-β can become a pro-oncogenic factor, promoting tumor progression, metastasis, and angiogenesis.^96^

In addition to promoting hepatocyte apoptosis/cell death, TGF-β signaling plays a crucial role in the metabolic dysregulation that characterizes ALD.

Ethanol induces a metabolic shift in hepatocytes by altering lipid synthesis, oxidation, and storage pathways^17^^,^^97^ and leads to lipid accumulation and metabolic dysregulation. Recent studies have shown that TGF-β signaling further exacerbates these pathologic changes in hepatocytes^74^^,^^98^ (eg, by impairing fatty acid oxidation).^60^

In line, transcriptomic analyses of primary murine hepatocytes exposed to both TGF-β and ethanol revealed an enhanced induction of genes involved in lipogenesis.^74^ These transcriptional changes were associated with impaired mitochondrial β-oxidation and a dysregulated oxidation-reduction environment, collectively promoting metabolic derangement.

TGF-β also down-regulates the alcohol-metabolizing enzyme ADH1 mRNA in cultured hepatocytes and liver tissue from TGF-β transgenic mice via the ALK5/Smad2/3 signaling branch, with Smad7 as a potent negative regulator.^74^ Furthermore, it was shown that ADH1 deficiency is a determining factor for the increased lipid accumulation and CYP2E1-dependent toxicity in liver cells on alcohol challenge. The down-regulation of ADH1 diverts ethanol metabolism toward the microsomal ethanol oxidation system, particularly through CYP2E1, which produces more ROS and exacerbates oxidative damage.^99^ This metabolic reprogramming fosters intracellular lipid accumulation, contributing to the development of macrovesicular steatosis—an early histologic feature of ALD.

Collectively, these findings indicate that TGF-β not only exacerbates hepatocyte apoptosis but also facilitates ethanol-induced steatotic transformation through metabolic rewiring as part of an oxidative stress repsonse.

## Debating the Role of Hepatocyte EMT in Fibrosis: Evidence for TGF-β–Mediated Pathogenic Plasticity in ALD

Several studies have highlighted the ability of TGF-β to induce partial EMT in hepatocytes, a process traditionally associated with fibrosis and cancer progression in the past.^27^^,^^100^, ^101^, ^102^, ^103^ EMT in hepatocytes is characterized by the loss of epithelial markers, such as E-cadherin, and the up-regulation of mesenchymal markers, like vimentin, fibronectin, and N-cadherin.^104^ This transformation enables hepatocytes to acquire a more migratory and invasive phenotype, potentially contributing to the progression of liver fibrosis and the development of HCC in the context of chronic liver injury.^27^^,^^59^^,^^105^, ^106^, ^107^ This concept was compromised by the work of Schwabe and his group.^108^ By deleting the TGFBR2 in liver epithelial cell injury, toxin-induced or biliary fibrosis, as well as hepatocarcinogenesis induced by diethylnitrosamine, was unaffected. From these data, they claim that TGF-β signaling in hepatocytes does not contribute to fibrosis or HCC formation. Although the data show that fibrosis and HCC can develop in a setting of a depleted canonical TGF-β pathway, it does not prove that, on the other hand, a functional TGF-β pathway that is activated in a cell context–dependent manner with respective responses, which may significantly contribute to disease development and progression, as impressively demonstrated in the work of Seki and coworkers^81^^,^^82^ above.

TGF-β–induced EMT in hepatocytes plays a key role in ALD, where chronic ethanol exposure drives sustained injury, inflammation, and fibrosis.^62^^,^^109^^,^^110^ In this context, TGF-β signaling promotes a partial EMT, shifting hepatocytes from a quiescent to a more motile and invasive phenotype. Rather than undergoing full mesenchymal conversion, these cells enhance fibrotic remodeling by promoting matrix deposition and recruiting fibrogenic cells.^17^^,^^62^^,^^111^

Ethanol-induced oxidative stress and inflammatory cytokines further potentiate EMT, thereby accelerating fibrosis progression.^17^^,^^60^^,^^101^

Both animal models and patient liver biopsies confirm elevated mesenchymal marker expression in hepatocytes, correlating with fibrosis severity in ALD.^17^^,^^110^ These findings highlight TGF-β–driven EMT as a central mechanism in ALD pathogenesis and fibrosis progression.

## From Chromatin to Cirrhosis: TGF-β Orchestrates Hepatocyte Failure via HNF4α Silencing in AH

Research on molecular drivers of severe AH and end-stage liver disease is hampered by the lack of suitable animal models.^10^ To gain some mechanistic insight into end-stage ALD, Bataller and colleagues^10^ conducted an in-depth analysis of RNA-sequencing data from livers of patients with varying ALD phenotypes. Severe AH was characterized by a marked reduction of liver-enriched transcription factors. Integrated gene expression analyses confirmed a global down-regulation of hepatocyte nuclear factor 4α (HNF4α) target genes, correlating with hepatocellular failure. A re-analysis of the RNA-sequencing data unfortunately was not possible because the raw data were not accessible. Epigenetic profiling revealed profound alterations in DNA methylation and chromatin remodeling in AH livers, particularly affecting HNF4α-dependent gene expression. Importantly, TGF-β emerged as a central upstream regulator driving the activation of the alternative HNF4α P2 promoter in hepatocytes, an event linked to the impaired metabolic and synthetic liver functions.

Down-regulation of HNF4α in patients with advanced decompensated cirrhosis is also described on the basis of immunohistochemical analysis of human samples.^112^

Interestingly, gene polymorphisms in liver-enriched transcription factors, including HNF4α, do not predispose to the development of AH, whereas AH livers are characterized by complex changes in DNA methylation state and chromatin remodeling in HNF4α-dependent genes.^10^^,^^113^ This human-based translational study further detected that the development of hepatocellular failure in patients with AH is characterized by a dramatic decrease in HNF4α-dependent gene expression. In contrast, patients with compensated cirrhosis and preserved synthetic function did not have a functional HNF4α deficiency. In line, it was demonstrated that re-expression of HNF4a led hepatocytes to regain function *in vitro* as well as *in vivo*.^114^

Together, these findings support the hypothesis that defective HNF4α expression or function underlies hepatocellular dysfunction in AH and possibly in other forms of decompensated liver disease. Targeting TGF-β signaling and epigenetic modifiers of HNF4α-dependent gene expression are suggested as a promising therapeutic strategy to restore hepatocellular function in patients with AH. Future analysis of transcription factor activity in AH should comparatively include compensated as well as decompensated patients to better understand the specificity of these findings and mechanisms of liver failure.

## From Alcohol to Fibrosis: TGF-β as a Master Regulator of HSC Activation

In CLD, including ALD, TGF-β exerts its profibrogenic effects primarily by activating HSCs.^65^^,^^66^ On activation by TGF-β, HSCs transdifferentiate into myofibroblasts, leading to excessive ECM deposition and the development of fibrosis.^67^

Thus, the TGF-β/Smad pathway is a central mediator of HSC activation and fibrogenesis in ALD. In HSCs, canonical TGF-β signaling regulates expression of fibrogenic genes, such as collagens (*COL1A1*, *COL3A1*) and α-smooth muscle actin (*ACTA2*).^115^^,^^116^ Ethanol sustains this pathway's activation, enhancing ECM accumulation.^62^^,^^117^

Ethanol-induced HSC activation also involves crosstalk with other profibrotic pathways, including NF-κB, p38 MAPK, and JNK, which synergize with TGF-β signaling to amplify fibrogenesis.

Additionally, in mice treated with ethanol for up to 16 weeks, it was shown that bone marrow–derived cells are recruited to the liver and can become activated HSCs expressing α-smooth muscle actin (α-SMA) and TGF-β, suggesting that chronic alcohol consumption stimulated the recruitment of activated HSCs from the bone marrow to the liver.^118^ However, these data were not supported by others, and the concept is still controversially discussed, also in the context of other CLD etiologies.^119^, ^120^, ^121^, ^122^

Ethanol metabolism results in the accumulation of acetaldehyde, a highly reactive metabolite that not only induces oxidative stress but also promotes the activation of latent TGF-β (LTGF-β) and enhances the expression of its type II receptor in HSCs,^123^^,^^124^ therewith amplifying autocrine and paracrine TGF-β signaling and reinforcing HSC activation and ECM production.

Thus, alcohol, acetaldehyde, and LPS have direct and indirect effects on HSCs, and all induce expression of TGF-β.^7^^,^^11^^,^^99^^,^^123^ LPS, which is elevated in the liver on alcohol consumption, can directly enhance stellate cell activation via up-regulation of TGF-β signaling and indirectly promotes stellate cell activation via activation of KCs to release profibrotic cytokines and chemokines.^30^

Although alcohol consumption causes hepatocyte damage, which leads to the release of a variety of mediators and the subsequent induction of HSC activation, as described above, its primary metabolite acetaldehyde also targets HSCs and up-regulates the expression of TGF-β and collagens in these cells and modulates TGF-β signaling.^7^^,^^99^^,^^123^^,^^125^^,^^126^

In detail, acetaldehyde induces the expression of TGF-β, promotes the activation of LTGF-β protein, and increases the expression of TGFBR2,^127^ thereby sensitizing HSCs to TGF-β–mediated responses. These effects occur within 6 to 12 hours of exposure and suggest that a substantial portion of acetaldehyde's fibrogenic activity is mediated indirectly through TGF-β1 signaling.^128^ However, acetaldehyde also elicits early fibrogenic responses independently of TGF-β1, particularly within the first 6 hours.^117^^,^^123^^,^^127^ These early effects are associated with phosphorylation of Smad3 and phosphatidylinositol 3-kinase–dependent pathways, without changes in Smad2 phosphorylation or TGF-β1 levels.^123^ These two temporally distinct mechanisms (ie, TGF-β–independent early activation and TGF-β–dependent late-phase responses) converge to enhance the expression of collagen α1(I) and α2(I) genes (*COL1A1*, *COL1A2*).^128^, ^129^, ^130^ Notably, combined treatment with acetaldehyde and TGF-β1 leads to additive up-regulation of collagen gene promoters, and TGF-β1 knockdown significantly reduces acetaldehyde-induced collagen α2(I) expression.^125^^,^^131^

At the transcriptional level, acetaldehyde activates multiple signaling pathways, including protein kinase C, JNK, and ERK, which converge on the transcription factor activator protein 1 (AP-1). AP-1, in turn, induces the expression of basic transcription element binding protein 1 (BTEB), a transcription factor that binds to the GC box of the TGFBR2 promoter and enhances receptor transcription. Binding of additional factors, such as nuclear factor I, to the α2(I) collagen promoter has also been implicated in acetaldehyde- and TGF-β–driven gene activation. Importantly, although the precise molecular mechanisms remain incompletely defined, it is proposed that acetaldehyde may either act directly on the TGF-β promoter or influence upstream transcriptional regulators that modulate its activity.^62^^,^^127^^,^^132^^,^^133^

Further supporting the complexity of this system, interactions between tumor-suppressor pathways and fibrogenic signaling have been described. For example, the Δ40p53α isoform can enhance TGF-β–induced collagen III expression by interacting with full-length p53 and phosphorylated Smad3, indicating crosstalk between p53 and TGF-β pathways during fibrogenesis.^134^

A central downstream effector of TGF-β in this context is connective tissue growth factor (CTGF).^135^^,^^136^ Ethanol and acetaldehyde both increase CTGF expression in human and murine HSCs, leading to enhanced production of α-SMA and collagens. This induction can be blocked by 4-methylpyrazole or N-acetylcysteine, indicating a role for oxidative metabolism and oxidation-reduction balance. CTGF promoter activity is up-regulated in a sustained manner by ethanol or TGF-β, and mutation of the Smad-binding or basal control element in the promoter significantly attenuates this effect. Silencing of TGF-β or CTGF via siRNA reduces ethanol- or acetaldehyde-induced mRNA and protein expression of CTGF, α-SMA, and collagen I in both LX-2 cells and primary mouse HSCs, confirming CTGF's pivotal role in this signaling axis.

Interstingly, TGF-β may also induce autophagy downstream of alcohol in HSCs by inhibiting Akt/mammalian target of rapamycin or activating ERK and JNK pathways, which subsequently leads to HSC activation and fibrosis.^137^, ^138^, ^139^, ^140^

Taken together, these findings underline the central importance of TGF-β in ethanol- and acetaldehyde-induced HSC activation and fibrogenesis. They reveal multiple regulatory nodes, including early TGF-β–independent mechanisms, late-phase TGF-β–dependent signaling, CTGF induction, and signaling and transcription factor crosstalk, that represent potential therapeutic targets for antifibrotic strategies in alcohol-related liver disease.

## miRNA Networks and Epigenetic Regulators Shape TGF-β Fibrotic Responses in ALD

Epigenetic and post-transcriptional mechanisms significantly influence the fibrogenic response to TGF-β in ALD. Epigenetic modifications, such as histone alterations and DNA methylation, may perpetuate the activated fibrotic state after ethanol cessation, thereby contributing to disease chronicity.^69^^,^^116^^,^^141^

Noncoding RNAs, including miRNAs and long noncoding RNAs, also modulate the expression of fibrogenic genes. Of particular importance is the let-7/Lin28 axis, which regulates HSC activation by targeting Smad proteins.^69^^,^^142^ In mouse models, chronic ethanol exposure down-regulated several let-7 family members, including let-7a and let-7b. Similarly, stimulation of human HSCs with LPS and TGF-β reduced let-7a and let-7b expression. Conversely, forced expression of these miRNAs suppressed HSC myofibroblastic activation, as indicated by decreased expression of α-SMA, collagen 1A1, tissue metalloprotease 1, and fibronectin 1. These findings highlight the let-7/Lin28 axis as a critical regulatory node in HSC activation and suggest potential therapeutic opportunities through modulation of miRNA networks in ALD.

In support of this, inhibition of miR-96 in combination with a Hedgehog pathway blocker attenuated liver injury and fibrosis in ALD models.^69^ miR-96 is up-regulated in ALD in mice and humans and promotes liver fibrosis by down-regulating Smad7 and Foxo1-3, enhancing TGF-β signaling. Inhibition of miR-96 in ethanol-treated hepatic cells, in turn, increased Smad7 expression and decreased TGF-β and Gli1, suggesting miR-96 as a potential therapeutic target in ALD.

Another profibrotic mechanism involves the engulfment of apoptotic bodies by HSCs, which leads to activation of LTGF-β and increased collagen synthesis, further amplifying fibrogenesis.^143^

Moreover, chronic alcohol feeding in wild-type mice led to elevated hepatic TGF-β mRNA levels, an effect absent in miR-155 knockout mice. This suggests a distinct, miR-155–dependent regulatory mechanism contributing to TGF-β up-regulation and fibrogenesis in ALD.^11^^,^^144^

Natural compounds, such as curcumin, suppress Smad activation and reduce HSC proliferation and ECM synthesis under alcohol exposure.^141^ Another natural potential therapeutic agent, butein, has demonstrated antifibrotic effects by suppressing ethanol-induced TGF-β expression and inhibiting downstream signaling cascades, including NF-κB and MAPKs, thereby attenuating HSC activation and ECM gene expression,^62^^,^^117^^,^^145^ thus providing potential therapeutic implications.

Shuai et al^146^ identified a novel, purinergic mechanisms by which ectonucleoside triphosphate diphosphohydrolase-1/ENTPD1 (CD39)–mediated ATP-adenosine signaling regulates TGF-β/Smad3 signaling during HSC-T6 activation and alcohol-related liver fibrosis. ATP is hydrolyzed to adenosine by different enzymes, including CD39. In ethanol plus carbon tetrachloride C57BL/6-treated mice as well as acetaldehyde-treated HSC-T6 hepatic stellate cells, pharmacologic interference experiments indicated that expression of fibrogenic factors is CD39 dependent. Moreover, blockade or silencing of CD39 could block the TGF-β/Smad3 pathway, alleviating alcoholic hepatic fibrosis.

These data indicate that inhibiting TGF-β signaling has shown therapeutic promise and highlight again the pathologic relevance of both canonical and noncanonical TGF-β signaling in ALD-related fibrosis.

## Sex-Specific Disparities in Alcohol-Induced Liver Fibrosis: TGF-β1/Smad3 Signaling and Hormonal Modulation Drive Male Susceptibility in Mice

Sex-related differences in ALD are obvious, presenting females more prone to adverse alcohol effects than males (previously comprehensively reviewed^147^). In contrast to this, Hong et al^148^ reported significant opposite sex differences associated with the TGF-β1/Smad3–signaling pathway in alcohol-related liver fibrosis. A total of 15 male and female C57BL/6N mice per group were fed a Lieber-DeCarli liquid diet for 8 weeks with or without two 5 g/kg alcohol gavages per week. HSC-T6 cells were treated with combinations of either estradiol plus ethanol, or testosterone plus ethanol. Compared with controls and the female model group, the male model group exhibited significantly increased glutamate-pyruvate transaminase (GPT), glutamic oxaloacetic transaminase (GOT), TNF-α, IL-6, and testosterone levels, a higher fibrosis rate, as well as more strongly induced TGF-β1, Smad3, and proliferating cell nuclear antigen expression levels, whereby estradiol levels and caspase-3 expression were significantly decreased. In HSC-T6, the apoptosis rate was higher in the estradiol/ethanol group than in the testosterone/ethanol group, although the latter exhibited increased TNF-α, IL-6, collagen-I, α-SMA, TGF-β1, Smad3, and proliferating cell nuclear antigen expression, and decreased caspase-3 expression. How these findings integrate in the higher sensitivity of females to alcohol consumption warrants further studies.

## TGF-β Drives LSEC Dysfunction in ALD: Diet-Dependent Capillarization, Cell Cycle Arrest, and Fibrogenic Reprogramming

In addition to its effects on hepatocytes and HSCs, TGF-β also plays a crucial role in modulating phenotype and function of LSECs during ALD. TGF-β inhibits LSEC proliferation, contributing to capillarization and impaired sinusoidal function, which exacerbates hepatic hypoxia and fibrosis in ALD models.^95^ Moreover, LSEC endogenous regulators, such as thioredoxin-interacting protein (TXNIP), can ameliorate ALD by promoting nitric oxide production, thereby counteracting the detrimental effects of TGF-β signaling on endothelial integrity.^149^ Using intragastric feeding in rats, the potent inhibitory effect on LSEC proliferation is mediated by TGF-β, which leads to altered vascular architecture and initiates fibrotic responses. These rats exhibit elevated levels of TGF-β in plasma and in the supernatant of cultured nonparenchymal cells, whereas TGF-β levels remained unchanged in animals fed saturated fat and ethanol or in control groups. Immunohistochemistry localized the up-regulation of TGF-β primarily to interstitial macrophages, likely KCs, in the ethanol/corn oil–fed animals. These observations suggest a diet-dependent up-regulation of hepatic TGF-β in response to alcohol. In detail, increased TGF-β in this model caused LSECs to arrest in the G_1_/S phase of the cell cycle, significantly reducing their proliferation and impairing endothelial regeneration. Cell cycle arrest further promotes sinusoidal capillarization, characterized by loss of fenestrae and basal lamina deposition, which disrupts molecular exchange between blood and hepatocytes, a hallmark of CLD. Ethanol combined with unsaturated fats strongly potentiates this inhibitory effect on endothelial turnover, whereas saturated fats increase endothelial proliferation. Sustained TGF-β exposure also induces endothelial cells to transdifferentiate into a smooth muscle–like phenotype with contractile properties, contributing to fibrosis. KCs produce TGF-β in response to activation by ethanol and polyunsaturated fats, rather than through increased Kupffer cell numbers. TGF-β binds with high affinity to receptors on LSECs. Through cell cycle arrest and phenotypic reprogramming, endothelial cells act as both targets and effectors in the fibrogenic cascade of ALD. Thus, TGF-β1 critically links endothelial dysfunction to fibrosis progression, with diet-alcohol interactions modulating disease severity and progression.^149^

Taken together, these findings highlight the contribution of TGF-β to LSEC dysfunction in ALD and suggest that preserving endothelial homeostasis may represent a novel antifibrotic strategy.

## KCs Fuel ALD Fibrosis: TGF-β Dominance, CD14-Dependent Inflammation, and miR-27a–Extracellular Vesicle Immune Reprogramming

KCs are among the first liver-resident immune cells to encounter alcohol-induced challenges, including gut-derived LPS. On activation, they initiate proinflammatory responses by releasing cytokines, such as TNF-α, IL-1β, and IL-6, various chemokines, and TGF-β, thereby promoting HSC activation.^117^^,^^150^^,^^151^

This activation is present in alcohol-induced liver injury and involves the pattern recognition receptor CD14, which is highly expressed on monocytes and macrophages and facilitates LPS binding. In a study using CD14 knockout and wild-type BALB/c mice fed with ethanol via enteral delivery for 4 weeks, the absence of CD14 prevented alcohol-induced increases in NF-κB, TNF-α, and TGF-β, indicating that CD14 is essential for Kupffer cell–mediated inflammatory signaling in ALD.^152^ TGF-β and LTGF-β were markedly elevated in ethanol-treated wild-type but not CD14-deficient mice.

Using isolated KCs from a rat model of alcohol-induced fibrosis, Tsukamoto and colleagues^153^^,^^154^ demonstrated that these cells are major producers of TGF-β and that their TGF-β is a primary driver of collagen expression in HSCs. Specifically, KC-derived TGF-β doubled HSC collagen production. Northern blot analysis confirmed increased TGF-β mRNA expression in KCs from ethanol-fed rats compared with controls. Although both groups secreted LTGF-β, only cells from ethanol-exposed rats released the active form, implicating KC-derived active TGF-β as a central mediator of HSC activation and fibrogenesis.^153^^,^^154^ To note here, a second wave of TGF-β production originates from activated HSCs themselves, perpetuating a fibrogenic feedback loop that has been observed in liver tissue of patients with ALD.^150^

Moreover, apoptotic bodies from damaged hepatocytes are phagocytosed by KCs, further increasing TGF-β production and reinforcing fibrotic signaling pathways.^117^

Notably, under certain conditions, KCs can also exert anti-inflammatory effects. On LPS exposure, they may release IL-10 and other regulatory cytokines, contributing to liver tolerance. Through these mechanisms, innate immunity may also suppress fibrogenesis by inducing apoptosis of activated HSCs or by interfering with TGF-β signaling.^117^^,^^151^

Interestingly, extracellular vesicles enriched in miR-27a are produced by alcohol-treated monocytes and can induce the polarization of naïve monocytes into M2 macrophages. These M2 macrophages are characterized by increased expression of surface markers, such as CD68, CD206 (mannose receptor), and CD163 (scavenger receptor), enhanced phagocytic activity, and secretion of anti-inflammatory cytokines, including IL-10 and TGF-β.^155^ Notably, circulating extracellular vesicles isolated from the plasma of patients with AH also showed high levels of miR-27a, suggesting that this mechanism is relevant *in vivo*. The study concluded that alcohol promotes extracellular vesicle production in monocytes and that extracellular vesicles serve as intercellular communicators, with miR-27a cargo directing monocyte polarization toward an anti-inflammatory M2 phenotype.

Chronic ethanol consumption induces oxidative stress, leading to increased TGF-β levels and suppression of interferon-γ signaling. This impairs natural killer cell–mediated cytotoxicity against activated HSCs, thereby promoting fibrogenesis.^156^, ^157^, ^158^, ^159^ In advanced stages of liver fibrosis, the antifibrotic effects of natural killer cells and interferon-γ are progressively diminished.^158^

In addition to weakening natural killer cell activity, TGF-β also fosters immune suppression by promoting the differentiation of regulatory T cells, which can dampen immune responses and impede the clearance of damaged hepatocytes, potentially sustaining liver injury in ALD.^156^^,^^158^, ^159^, ^160^, ^161^, ^162^

Furthermore, TGF-β cooperates with IL-6 to promote the differentiation of naïve CD4^+^ T cells into type 17 helper T cells, as shown by Nagy and colleagues^163^ and Veldhoen et al^164^ in 2006. This shift depletes regulatory T cells and enhances inflammation through IL-17A secretion by type 17 helper T cells, which recruits and activates neutrophils and macrophages. In patients with AH and cirrhosis, elevated plasma IL-17 levels and increased IL-17^+^ inflammatory infiltrates have been observed, with cell numbers correlating with disease severity.^165^ Although TGF-β is known to drive type 17 helper T cell differentiation *in vitro*, its specific involvement in this pathway in human ALD remains to be established.

## TGF-β at the Crossroads of Liver Regeneration and Fibrosis: Balancing Progenitor Cell Fate in End-Stage Liver Disease

In patients with end-stage liver disease (ie, decompensated cirrhosis, acute-on-chronic liver failure, acute liver failure, or severe AH), a strategy to survive for the patients is the functional replacement of the necrosis-based hepatocyte loss by cholangiocyte-based liver progenitor cells. Because of this, a massive proliferative activity of liver progenitor cells can be observed, which is also termed ductular reaction. In this setting, the second pathway of liver regeneration is initiated, leading to complete transdifferentiation of cholangiocytes to hepatocytes,^166^^,^^167^ or to provide liver functions faster, changing the transcriptional program to activate liver function genes.^167^, ^168^, ^169^, ^170^, ^171^, ^172^

Zooming into cellular events, hedgehog (Hh)–initiated pathways were found activated in these immature ductular cells in patients with ALD. Importantly, lobular accumulation of Hh-responsive liver progenitor cells was greater in patients with a discriminant function > 32, which is indicative for higher mortality. Hh signaling is also strongly induced in mice fed a high-fat diet plus alcohol and promotes hepatic accumulation of immature ductular cells (ductular reaction). These mice also expressed more TGF-β, which in this case promoted the viability of Hh-responsive immature liver cells and caused Hh ligand production in surviving mature hepatocytes.^173^ Tong et al^172^ recently performed an in-depth spatial transcriptomics analysis of liver tissues from patients with acute liver failure to delineate cell-cell communication with incoming and outgoing signals between liver progenitor cells (LPCs) and the environment. Multiple signals from surrounding macrophages and HSCS, including TGF-β, hepatocyte growth factor (HGF), and epidermal growth factor (EGF), could be identified. The effects of TGF-β were functionally tested in cultured LPCs and in 3,5-diethoxycarbonyl-1,4-dihydrocollidine–fed mice, which present with such aforementioned ductular reaction, but more as fibrogenesis-supporting mechanism in this case. In the cultured cells, TGF-β inhibits proliferation by impeding G_1_-S phase transition. In the mouse model as well, ectopic Smad7 expression increased LPC proliferation. This is also supported from the above described investigation with liver epithelial deletion of TGFBR2, where Schwabe and coworkers^108^ showed an increase in tumor number and a shift from HCC to cholangiocarcinoma in mice with a concomitant hepatic deletion of PTEN. Surprisingly, both hepatocyte- and cholangiocyte-specific deletion of PTEN and TGFBR2 promoted the development of cholangiocarcinoma. Their data further suggest that cholangiocarcinoma arise from hepatocyte-derived cholangiocytes, whereby PTEN deletion resulted in up-regulation of TGFBR2, and deletion of TGFBR2 increased cholangiocyte proliferation, indicating that the main function of epithelial TGFBR2 is to restrict cholangiocyte proliferation.^108^

In the patients with ALF, however, extensive LPC proliferation was observed despite the presence of TGF-β–phosphorylated SMAD2 signaling in the activated LPCs.^172^ In the same cells, proliferative phosphorylated HGF receptor (MET), phosphorylated STAT3, phosphorylated epidermal growth factor receptor, and phosphorylated ERK signaling pathways are activated by HGF and EGF ligands, which are able to interfere with the antiproliferative TGF-β signaling. Beyond acting as mitogens, HGF and EGF regulate master hepatocyte genes (eg, HNF4α), whereby TGF-β–activated SMADs are now required for this functional transdifferentiation, which is in line with the above findings of.^173^

## Conclusion and Future Perspective

In this review, the impact of TGF-β signaling in ALD is summarized, putting special emphasis on its cell type–specific effects in the liver. Cell line, mouse-derived, and human-derived data were included. It is obvious that TGF-β signaling is regulated by various intersecting mechanisms and is itself orchestrating a wide range of context-specific effects. Thereby, intervening with TGF-β signaling remains complex. Liver injury of diverse etiologies, including ALD, unleashes active TGF-β ligands that engage virtually all hepatic cell types. Although TGF-β has long been recognized as a central profibrogenic cytokine and numerous therapeutic strategies are in development, translation from animal models to the clinic of CLD, including ALD, has remained elusive. A key challenge lies in the stark temporal and biological differences between experimental models, where fibrosis and inflammation evolve within weeks, and human disease, which unfolds over decades and passes through multiple phases. Across these stages, TGF-β exerts opposing functions as promoting epithelial cell death and oxidative stress, driving myofibroblast activation and fibrogenesis, restraining hepatocyte regeneration, and shaping immune responses yet also limiting premalignant growth and influencing the tumor microenvironment by modulating fibroblasts, angiogenesis, and EMT. This functional duality underlines the complexity of targeting TGF-β in CLD. Future strategies will require the careful definition of therapeutic windows, cell type specificity, and selective interference with detrimental signaling branches, a task that demands further substantial mechanistic insight from basic research.

## Disclosure Statement

None declared.
